# Barriers to Utilizing Social Media Platforms in Emergency Medicine Residency Programs

**DOI:** 10.7759/cureus.5856

**Published:** 2019-10-07

**Authors:** Jay Khadpe, Manpreet Singh, Zachary Repanshek, Emily Brumfield, Faheem Guirgis, Colleen Kalynych, Carmen Smotherman, Michelle Lott, Abbas Husain

**Affiliations:** 1 Emergency Medicine, University of Florida College of Medicine - Jacksonville, Jacksonville, USA; 2 Emergency Medicine, Harbor–University of California at Los Angeles (UCLA) Medical Center, Torrance, USA; 3 Emergency Medicine, Lewis Katz School of Medicine at Temple University, Philadelphia, USA; 4 Emergency Medicine, Ochsner Clinic Foundation, New Orleans, USA; 5 Emergency Medicine, University of Florida Health Jacksonville, Jacksonville, USA; 6 Miscellaneous, University of Florida College of Medicine - Jacksonville, Jacksonville, USA; 7 Emergency Medicine, Staten Island University Hospital, Staten Island, USA

**Keywords:** emergency medicine, medical education, social media, barriers, graduate medical education

## Abstract

Background

Residency programs seek to incorporate various social media (SoMe) platforms into their educational curricula, yet little is known regarding the potential roadblocks towards implementation. Our objective was to assess the current utilization of SoMe platforms and identify common barriers to implementation by emergency medicine (EM) residency programs.

Methods

Members of the Council of Emergency Medicine Residency Directors (CORD) Information Technology (IT) Committee developed an anonymous survey distributed to representatives from EM residency programs using the “CORD Community” internet forum. Descriptive statistics including percentages for numerical data as well as Fisher’s exact test for categorical data were used to report results.

Results

We received 116 individual responses from faculty, fellows, and residents of EM residency programs. The most common institutional, departmental, technological and knowledge barriers identified were restricted access to blogs (12.9%), insufficient protected time (17.2%), insufficient IT support to host the platform (16.4%), and a lack of knowledge among faculty of how to utilize blogs (23.3%) respectively.

Ten respondents (8.6%) reported that their programs had not attempted to utilize any SoMe platforms. Community-based programs and smaller programs (<24 residents) were significantly more likely to identify barriers to SoMo use among this cohort.

Conclusion

Utilization of SoMe platforms for resident education by EM residency programs is increasingly common, but significant obstacles exist on many levels that prevent programs from leveraging these innovations for knowledge translation. This is particularly common for community-based and small residency programs. Awareness of these common barriers will allow institutions and programs to better anticipate and design solutions to overcome these obstacles.

## Introduction

The rise of social media (SoMe) as a means to transfer knowledge and facilitate communication birthed the development of the free open access medical education (FOAM) movement. FOAM is defined as an evolving collection of resources, a community, and an ethos [1]. Organizations, such as the Council of Emergency Medicine Residency Directors (CORD), that are charged with leading the educational paradigm for medical specialties, have published guidelines and best practices on how to implement these largely SoMe-based resources [2-3]. Some individual residency programs have followed suit by integrating their own self-produced FOAM resources into their curricula [4].

As the popularity of these technologies for medical education has grown, so too has the variety of applications within training programs. Twitter® is used by numerous emergency medicine (EM) residencies to disseminate educational content from weekly conferences [5]. Journal clubs can now be attended virtually using video-based platforms with Twitter® and blog integration [6]. The interpretation of electrocardiograms can be taught using social networking sites [7]. Even an entire encyclopedia of core EM knowledge can be shared and continuously updated using a wiki-based platform [8]. The possibilities of the integration between SoMe and medical education are seemingly endless.

Academic faculty are being charged with leading efforts to create curricula around the use of SoMe to better reach and engage today’s learners. As such, there is increasing interest in how such scholarly work should be assessed by Promotion and Tenure Committees [9-11]. In the future, utilization of SoMe may be an important component of an educational portfolio.

However, not all programs have been equally successful at implementing SoMe platforms into their residency education. Potential barriers exist at the institutional, departmental, and individual levels, which may prevent the utilization of these technologies. Understanding what specific barriers most commonly interfere with the implementation of SoMe platforms for educational purposes may help organizations to anticipate and target solutions for implementation.

The objective of this study was to assess the current utilization of SoMe platforms by EM residency programs and identify common barriers to their implementation.

## Materials and methods

CORD is a scientific and educational organization with member programs of all categorical EM residency training programs accredited by the Accreditation Council for Graduate Medical Education or by the American Osteopathic Association [12]. Members of the CORD Information Technology (IT) Committee established face validity for the survey instrument's language and consensus questions (refer Table 6 in Appendices). The survey was piloted at the author's individual sites for content and response process validity from faculty associated with the author's institutions. The University of Florida Institutional Review Board deemed this study exempt. The survey was anonymous and respondents were only asked demographical data with respect to their role in their member program (program director (PD), associate/assistant PD, clerkship director (CD), associate/assistant clerkship director, core faculty, other faculty, fellow, or resident), length of training format (three or four years), setting of their member program (academic, community, county, or hybrid), and size of the residency training program (small, medium, or large). The survey was designed in REDCap® (hosted by the University of Florida) a secure, web-based application designed to support data for research studies using a branching logic format that allowed respondents to indicate which SoMe platforms they were currently or previously attempted to utilize for residency education [13]. The survey only queried the respondent regarding selected platforms with respect to what barriers they had encountered. Potential barriers to implementation of SoMe platforms were categorized into institutional, departmental, technological, and knowledge/expertise.

The target audience for the survey was members of EM residency programs who currently or had previously attempted to implement SoMe platforms for the purpose of resident education. In an effort to reach as many representatives from EM residencies as possible, the survey was distributed through the “CORD Community” internet forum, which includes PDs, faculty, program coordinators, and residents. A post was made on the forum containing a link to the survey with two subsequent reminders to attempt to increase the response rate. The post also invited members to share the link with other representatives from their program who would be included in the target audience.

Data collection took place December 2017 through February 2018. The authors analyzed the data using the built-in tools from REDCap® and Microsoft Excel®. Descriptive statistics including frequencies and percentages were used to report the results for numerical data as well as Fisher’s exact test for categorical data from a group comparison of the barriers identified to the programs’ self-identified setting and size.

## Results

We received 116 responses from members of EM residency programs. Of those, PDs accounted for 16%, Associate/Assistant PDs 28%, CDs 5%, Core Faculty 13%, Other Faculty 5%, Fellows 4%, and Residents 28% of the responses. Sixty-nine percent of the responses came from the residency programs with a three-year format and 31% from those with a four-year format. Respondents described their residency program setting as academic (32%), community (16%), county (25%), and hybrid (26%). With regard to the size of the programs, small (≤24 residents) accounted for 16%, medium (25-39 residents) 33%, and large (>39 residents) 51%.

Audio-based platforms (e.g., podcasts) and blogs were the most common SoMe platforms utilized with more than half of respondents indicating they were currently or had previously attempted them for residency education (Table 1). The least utilized were photo-based (e.g., Instagram®) platforms with only 12.9% of respondents indicating they had implemented them for resident education and only 8.6% indicated they had not used any of the SoMe platforms for residency education purposes.

**Table 1:** Utilization of social media platforms for residency education

Platform	Respondents Use (%)
Blogs (e.g., WordPress, Blogger)	61 (52.6%)
Microblogging (e.g., Twitter, Tumblr)	53 (45.7%)
Social networking (e.g., Facebook, Google+, LinkedIn)	44 (37.9%)
Photo-based (e.g., Instagram, Snapchat, Pinterest)	15 (12.9%)
Audio-based (e.g., Podcasts)	65 (56.0%)
Video-based (e.g., Vodcasts, YouTube, Vimeo)	57 (49.1%)

Responses regarding institutional, departmental, technological, and knowledge/expertise barriers to the implementation of SoMe into residency educational programs are described in Tables 2-5. Table 2 describes responses regarding three potential institutional barriers: institution does not allow platform to be administered, institution imposes undue regulatory burden, and institution restricts access on campus. Table 3 describes responses to seven potential departmental barriers: department chair does not support/believe in the use of SoMe, insufficient faculty interest, insufficient resident interest, department will not provide adequate funds, department does not consider SoMe beneficial, insufficient protected time, and fear of professionalism violations. Table 4 describes responses to three potential technological barriers: insufficient IT support in hosting platforms, insufficient wireless internet access on campus, and insufficient support, space, and/or funding for necessary equipment. Table 5 describes responses to four potential knowledge and/or expertise barriers: faculty lack knowledge of how to use SoMe for education, residents lack knowledge of how to use SoMe for education, program lacks sufficient faculty leadership with expertise to implement, and residents do not have the ability to critically appraise for quality and accuracy.

**Table 2:** Institutional barriers with regard to implementation of social media platforms into the educational program

Platform	Not allowed	Regulatory burden	Restricts access	None
Blogs	11 (9.5%)	12 (10.3%)	15 (12.9%)	26 (22.4%)
Microblogging	5 (4.3%)	8 (6.9%)	14 (12.1%)	23 (19.8%)
Social networking	7 (6.0%)	9 (7.8%)	12 (10.3%)	11 (9.5%)
Photo-based	1 (0.9%)	1 (0.9%)	4 (3.5%)	2 (1.7%)
Audio-based	0	4 (3.5%)	8 (6.9%)	30 (25.9%)
Video-based	3 (2.6%)	6 (5.2%)	14 (12.1%)	21 (18.1%)
Other	2 (1.7%)	3 (2.6%)	2 (1.7%)	3 (2.6%)

**Table 3:** Departmental barriers with regard to implementation of social media platforms into the educational program

Platform	Department Chair	Faculty Interest	Resident Interest	Departmental Funds	Platform not considered beneficial	Protected Time	Fear of violations	None
Blogs	2 (1.7%)	19 (16.4%)	9 (7.8%)	7 (6.0%)	2 (1.7%)	20 (17.2%)	14 (12.1%)	17 (14.7%)
Microblogging	0	14 (12.1%)	10 (8.6%)	2 (1.7%)	1 (0.9%)	12 (12.1%)	13 (11.2%)	13 (11.2%)
Social networking	1 (0.9%)	10 (8.6%)	7 (6.0%)	2 (1.7%)	2 (1.7%)	9 (7.8%)	15 (12.9%)	7 (6.0%)
Photo-based	0	2 (1.7%)	2 (1.7%)	1 (0.9%)	1 (0.9%)	1 (0.9%)	4 (3.5%)	2 (1.7%)
Audio-based	3 (2.6%)	12 (10.3%)	2 (1.7%)	5 (4.3%)	3 (2.6%)	14 (12.1%)	7 (6.0%)	20 (17.2%)
Video-based	2 (1.7%)	7 (6.0%)	2 (1.7%)	4 (3.5%)	3 (2.6%)	12 (12.1%)	7 (6.0%)	17 (14.7%)
Other	0	4 (3.5%)	1 (0.9%)	1 (0.9%)	0	3 (2.6%)	1 (0.9%)	2 (1.7%)

**Table 4:** Technological barriers with regard to implementation of social media platforms into the educational program

Platform	IT Support	Internet Access	Equipment	None
Blogs	19 (16.4%)	4 (3.5%)	14 (12.1%)	23 (19.8%)
Microblogging	10 (8.6%)	4 (3.5%)	13 (11.2%)	19 (16.4%)
Social networking	5 (4.3%)	4 (3.5%)	12 (10.3%)	15 (12.9%)
Photo-based	1 (0.9%)	1 (0.9%)	1 (0.9%)	4 (3.5%)
Audio-based	9 (7.8%)	1 (0.9%)	13 (11.2%)	23 (19.8%)
Video-based	10 (8.6%)	3 (2.6%)	10 (8.6%)	24 (20.7%)
Other	1 (0.9%)	0	0	5 (4.3%)

**Table 5:** Knowledge and/or expertise barriers with regard to implementation of social media platforms into the educational program

Platform	Faculty knowledge	Resident knowledge	Faculty leadership/expertise	Quality appraisal	None
Blogs	27 (23.3%)	12 (10.3%)	21 (18.1%)	14 (12.1%)	16 (13.8%)
Microblogging	22 (19.0%)	10 (8.6%)	14 (12.1%)	9 (7.8%)	14 (12.1%)
Social networking	11 (9.5%)	7 (6.0%)	7 (6.0%)	12 (10.3%)	11 (9.5%)
Photo-based	2 (1.7%)	2 (1.7%)	2 (1.7%)	2 (1.7%)	2 (1.7%)
Audio-based	13 (11.2%)	5 (4.3%)	10 (8.6%)	9 (7.8%)	23 (19.8%)
Video-based	13 (11.2%)	6 (5.2%)	12 (10.3%)	10 (8.6%)	21 (18.1%)
Other	3 (2.6%)	2 (1.7%)	0	1 (0.9%)	1 (0.9%)

Among those programs that had not attempted to utilize any SoMe platforms (8.6%), community-based programs were significantly more likely to identify barriers compared to other program settings including insufficient faculty knowledge (p<0.001), fear of a professionalism violation (p<0.001), insufficient faculty interest (p=0.012), lack of institutional support (p<0.001), lack of technological support (p=0.012), lack of funding (p=0.012), and lack of protected time (p=0.026). With regard to size of programs, small programs (≤ 24 residents) were significantly more likely to identify barriers among those programs that had not utilized any SoMe platforms including insufficient faculty knowledge (p=0.002), fear of a professionalism violation (p<0.001), insufficient faculty interest (p=0.014), insufficient resident interest (p=0.026), lack of institutional support (p=0.002), lack of technological support (p<0.001), and lack of funding (p<0.001).

## Discussion

The results of our survey support the growing interest and use of SoMe platforms in graduate medical education (GME) [4,14-16]. Of those responding to our survey, 91% were currently utilizing or had previously attempted to utilize SoMe for residency education, with many indicating use of multiple platforms, suggesting that they are becoming commonplace in the realm of GME. However, prior to this study, it was not known what barriers exist to the implementation of SoMe platforms at EM residency programs across the country. Our survey results show several barriers exist on many levels within programs that impede the use of SoMe for education.

At the institutional level, we found restricting access to blogs and microblogs on campus to be the highest reported barriers. In a recent meta-analysis of SoMe use by GME programs, it was found that blogs and microblogs (e.g., Twitter®) were the most commonly used platforms for resident knowledge and education along with podcasts [17]. For those institutions where such barriers exist, it is imperative that leadership at all levels work toward identifying solutions so that programs can take advantage of these platforms and engage their learners with modern educational techniques.

We found that a lack of protected time, lack of faculty interest, and fear of professionalism violations were the most common barriers identified at the departmental level. To our knowledge, there is no available data on protected time for faculty to implement SoMe for residency education. In a previous survey, it was found that interest, or lack thereof, among residents and faculty to use SoMe for professional purposes was similar with 41.3% indicating a “low” or “very low” interest [18]. The concerns regarding professionalism and the use of SoMe have been well studied with many proposed guidelines for responsible use [19-22]. In one survey of EM residents and faculty, it was shown that high-risk professionalism events related to SoMe use are common within training programs which may explain why some programs are reluctant to encourage SoMe use for educational purposes [23]. Such events may be avoidable by utilizing previously published guidance on implementation strategies and best practices from CORD working groups with particular attention to professionalism and privacy concerns [2-3]. Understanding the underlying causes of these barriers will be key to developing solutions at the departmental level to utilize SoMe platforms.

With regard to potential technological barriers, we found the most common to be insufficient support, space, and/or funding for necessary equipment. With the rise of electronic medical records, there has been increasing emphasis on privacy and security concerns, which may have led academic institutions to a more closed and isolated strategy when first constructing their networking infrastructure [24]. However, this must be balanced with the academic mission and the need to incorporate education through SoMe within GME programs. Leadership at the program, departmental, and institutional levels must collaborate with IT personnel to develop strategies to provide secure access to private patient-level data while at the same time support the open sharing of medical knowledge using SoMe among their faculty and trainees.

Our study identified a lack of faculty knowledge of how to utilize several platforms as the most common barrier concerning knowledge or expertise with the use of SoMe. Programs that lack faculty leadership with expertise in SoMe should take advantage of opportunities to collaborate with faculty from other programs who have successfully implemented SoMe. The CORD IT Committee offers free consultation services to member programs who request expertise in using these resources. Additionally, programs may choose to support interested faculty to attend faculty development courses with an emphasis on SoMe and education. Blogs were noted to have the most knowledge/expertise barriers with lack of resident ability to appraise quality/accuracy only eclipsed by lack of faculty interest and leadership. This highlights the importance of protecting time and fostering these interests and skills in today's and tomorrow's educational leaders. Education concerning the appraisal of any medical education resource regardless of whether it is published in a journal or on a blog is a necessary component of every GME curriculum. There has been increasing focus on how best to appraise the quality of FOAM resources, yielding best practices that can be incorporated into the residency curriculum to overcome concerns with regard to the assessment of quality and accuracy [25-28].

Among those who responded that they had not attempted to utilize any SoMe platforms for educational purposes, we found that community-based programs and small programs (≤ 24 residents) were more likely to identify barriers to implementation of SoMe. This suggests that programs of these types may be at a particular disadvantage when it comes to trying to initiate curricula utilizing SoMe platforms. As the number of training programs continues to grow, many new smaller and/or community-based programs will need to anticipate these obstacles and plan solutions which may involve leveraging relationships at the local, regional, or national level including CORD [29].

Our study has identified common barriers to implementing SoMe platforms for educational purposes for EM residency programs. We also have identified that programs based at community hospitals and smaller sized residencies may face greater barriers with respect to these technologies. Some potential strategies for overcoming these barriers already exist but in other cases, it may be necessary for educators to continue to work together to identify best practices and solutions so that all learners might benefit from these innovations.

## Conclusions

SoMe is becoming an essential component of EM residency curricula. Organizations with a mission to lead the advancement of EM education need to identify those barriers that exist to stifle the development of innovations using SoMe platforms. Our study represents the first effort to identify specific barriers at the institutional, departmental, and individual level to utilizing SoMe for residency education. Our hope is that this will lead to targeted efforts to alleviate those barriers through a collaborative effort among the EM education and greater GME community.

## Appendices

**Table 6:** Survey instrument

We ask your assistance in order to learn more about the barriers to utilizing social media platforms in resident education. The survey will take about 2-5 minutes. We will not capture any PHI, all data is collected anonymously. The IRB has approved this as an exempt study and by taking this survey, you are agreeing to participate and providing consent. Thank you!	
Program Information	
What is your current role?	Program Director
	Associate/Assistant Program Director
	Clerkship Director
	Associate/Assistant Clerkship Director
	Core Faculty
	Other Faculty
	Fellow
	Resident
What is your residency program format?	3 years
	4 years
What best describes your residency program setting?	Academic
	Community
	County
	Hybrid
What is the size of your residency program?	Small: less than or equal to 24
	Medium: 25-39
	Large: >39
Are you currently or have you in the past attempted to utilize any of the social media platforms listed for the purposes of resident education? Check all that apply. For each selected platform, you will be asked specific questions about that platform.	Blogs (e.g., WordPress, Blogger)
	Microblogging (e.g., Twitter, Tumblr)
	Social networking (e.g., Facebook, Google+, LinkedIn)
	Photo-based (e.g., Instagram, Snapchat, Pinterest)
	Audio-based (e.g., Podcasts)
	Video-based (e.g., Vodcasts, YouTube, Vimeo)
	Other
	No
Blogs	
Which of the following INSTITUTIONAL barriers has your program experienced with regard to implementation of BLOGS into your educational program? Check all that apply:	Institution does not allow for programs to administer blogging platforms (e.g., legal department restriction)
	Institution imposes undue regulatory burden on the administration of blogging platforms
	Institution restricts access to blogging sites on campus internet (e.g., firewall limits computer access)
	None
Which of the following DEPARTMENTAL barriers has your program experienced with regard to implementation of BLOGS into your educational program? Check all that apply:	Department chair does not support or believe in the use of blogging for educational purposes
	Insufficient faculty interest in utilizing blogging for educational purposes
	Insufficient resident interest in utilizing social media for educational purposes
	Department will not provide adequate funds to support residency-based blogs
	Department does not consider blogs beneficial to the educational curriculum
	Insufficient protected time to develop educational curricula using blogs
	Fear of professionalism violations by participating residents and/or faculty
	None
Which of the following TECHNOLOGICAL barriers has your program experienced with regard to implementation of BLOGS into your educational program? Check all that apply:	Insufficient IT support in hosting blogs
	Insufficient wireless internet access on campus
	Insufficient support, space, and/or funding for necessary equipment (e.g., computer, microphone, video camera, audio/video editing software)
	None
Which of the following KNOWLEDGE and/or EXPERTISE barriers has your program experienced with regard to implementation of BLOGS into your educational program? Check all that apply:	Faculty lack knowledge of how to use blogs for educational purposes
	Residents lack knowledge of how to use blogs for educational purposes
	Program lacks sufficient faculty leadership with expertise in implementing blogs for educational purposes
	Residents do not have the ability to critically appraise blogs for quality and accuracy
	None
Are there any other barriers regarding the implementation of BLOGS that you have encountered?	
MICROBLOGGING	
Which of the following INSTITUTIONAL barriers has your program experienced with regard to implementation of MICROBLOGGING into your educational program? Check all that apply:	Institution does not allow for programs to administer microblogging platforms (e.g., legal department restriction)
	Institution imposes undue regulatory burden on the administration of microblogging platforms
	Institution restricts access to microblogging sites on campus internet (e.g., firewall limits computer access)
	None
Which of the following DEPARTMENTAL barriers has your program experienced with regard to implementation of MICROBLOGGING into your educational program? Check all that apply:	Department chair does not support or believe in the use of microblogging for educational purposes
	Insufficient faculty interest in utilizing microblogging for educational purposes
	Insufficient resident interest in utilizing social media for educational purposes
	Department will not provide adequate funds to support residency-based microblogging platforms
	Department does not consider microblogging to be beneficial to the educational curriculum
	Insufficient protected time to develop educational curricula using microblogging
	Fear of professionalism violations by participating residents and/or faculty
	None
Which of the following TECHNOLOGICAL barriers has your program experienced with regard to implementation of MICROBLOGGING into your educational program? Check all that apply:	Insufficient IT support in hosting microblogging platforms
	Insufficient wireless internet access on campus
	Insufficient support, space, and/or funding for necessary equipment (e.g., computer, microphone, video camera, audio/video editing software)
	None
Which of the following KNOWLEDGE and/or EXPERTISE barriers has your program experienced with regard to implementation of MICROBLOGGING into your educational program? Check all that apply:	Faculty lack knowledge of how to use microblogging for educational purposes
	Residents lack knowledge of how to use microblogging for educational purposes
	Program lacks sufficient faculty leadership with expertise in implementing microblogging for educational purposes
	Residents do not have the ability to critically appraise microblogging for quality and accuracy
	None
Are there any other barriers regarding the implementation of MICROBLOGGING that you have encountered?	
SOCIAL NETWORKING	
Which of the following INSTITUTIONAL barriers has your program experienced with regard to implementation of SOCIAL NETWORKING into your educational program? Check all that apply:	Institution does not allow for programs to administer social networking platforms (e.g., legal department restriction)
	Institution imposes undue regulatory burden on the administration of social networking platforms
	Institution restricts access to social networking sites on campus internet (e.g., firewall limits computer access)
	None
Which of the following DEPARTMENTAL barriers has your program experienced with regard to implementation of SOCIAL NETWORKING into your educational program? Check all that apply:	Department chair does not support or believe in the use of social networking platforms for educational purposes
	Insufficient faculty interest in utilizing social networking for educational purposes
	Insufficient resident interest in utilizing social networking for educational purposes
	Department will not provide adequate funds to support residency-based social networking platforms
	Department does not consider social networking platforms beneficial to educational curriculum
	Insufficient protected time to develop educational curricula using social networking platforms
	Fear of professionalism violations by participating residents and/or faculty
	None
Which of the following TECHNOLOGICAL barriers has your program experienced with regard to implementation of SOCIAL NETWORKING into your educational program? Check all that apply:	Insufficient IT support in hosting social networking platforms
	Insufficient wireless internet access on campus
	Insufficient support, space, and/or funding for necessary equipment (e.g., computer, microphone, video camera, audio/video editing software)
	None
Which of the following KNOWLEDGE and/or EXPERTISE barriers has your program experienced with regard to implementation of SOCIAL NETWORKING into your educational program? Check all that apply:	Faculty lack knowledge of how to use social networking platforms for educational purposes
	Residents lack knowledge of how to use social networking platforms for educational purposes
	Program lacks sufficient faculty leadership with expertise in implementing social networking platforms for educational purposes
	Residents do not have the ability to critically appraise social networking platforms for quality and accuracy
	None
Are there any other barriers regarding the implementation of SOCIAL NETWORKING that you have encountered?	
PHOTO-BASED SOCIAL MEDIA PLATFORMS	
Which of the following INSTITUTIONAL barriers has your program experienced with regard to implementation of PHOTO-BASED social media platforms into your educational program? Check all that apply:	Institution does not allow for programs to administer photo-based social media platforms (e.g., legal department restriction)
	Institution imposes undue regulatory burden on the administration of photo-based social media platforms
	Institution restricts access to photo-based social media sites on campus internet (i.e., firewall limits computer access)
	None
Which of the following DEPARTMENTAL barriers has your program experienced with regard to implementation of PHOTO-BASED social media into your educational program? Check all that apply:	Department chair does not support or believe in the use of photo-based social media for educational purposes
	Insufficient faculty interest in utilizing photo-based social media for educational purposes
	Insufficient resident interest in utilizing photo-based social media for educational purposes
	Department will not provide adequate funds to support residency-based photo-based social media platforms
	Department does not consider photo-based social media platform beneficial to educational curriculum
	Insufficient protected time to develop educational curricula using photo-based social media platform
	Fear of professionalism violations by participating residents and/or faculty
	None
Which of the following TECHNOLOGICAL barriers has your program experienced with regard to implementation of PHOTO-BASED social media into your educational program? Check all that apply:	Insufficient IT support in hosting photo-based social media platforms
	Insufficient wireless internet access on campus
	Insufficient support, space, and/or funding for necessary equipment (e.g., computer, microphone, video camera, audio/video editing software)
	None
Which of the following KNOWLEDGE and/or EXPERTISE barriers has your program experienced with regard to implementation of PHOTO-BASED social media into your educational program? Check all that apply:	Faculty lack knowledge of how to use photo-based social media platforms for educational purposes
	Residents lack knowledge of how to use photo-based social media platforms for educational purposes
	Program lacks sufficient faculty leadership with expertise in implementing photo-based social media platforms for educational purposes
	Residents do not have the ability to critically appraise photo-based social media platforms for quality and accuracy
	None
Are there any other barriers regarding the implementation of PHOTO-BASED social media that you have encountered?	
AUDIO-BASED SOCIAL MEDIA PLATFORMS	
Which of the following INSTITUTIONAL barriers has your program experienced with regard to implementation of AUDIO-BASED social media into your educational program? Check all that apply:	Institution does not allow for programs to administer audio-based social media platforms (e.g., legal department restriction, etc.)
	Institution imposes undue regulatory burden on the administration of audio-based social media platforms
	Institution restricts access to audio-based social media sites on campus internet (e.g., firewall limits computer access)
	None
Which of the following DEPARTMENTAL barriers has your program experienced with regard to implementation of AUDIO-BASED social media into your educational program? Check all that apply:	Department chair does not support or believe in the use of audio-based social media for educational purposes
	Insufficient faculty interest in utilizing audio-based social media for educational purposes
	Insufficient resident interest in utilizing audio-based social media for educational purposes
	Department will not provide adequate funds to support residency-based audio-based social media platforms
	Department does not consider audio-based social media platforms beneficial to the educational curriculum
	Insufficient protected time to develop educational curricula using audio-based social media platforms
	Fear of professionalism violations by participating residents and/or faculty
	None
Which of the following TECHNOLOGICAL barriers has your program experienced with regard to implementation of AUDIO-BASED social media into your educational program? Check all that apply:	Insufficient IT support in hosting audio-based social media platform
	Insufficient wireless internet access on campus
	Insufficient support, space, and/or funding for necessary equipment (e.g., computer, microphone, video camera, audio/video editing software)
	None
Which of the following KNOWLEDGE and/or EXPERTISE barriers has your program experienced with regard to implementation of AUDIO-BASED social media into your educational program? Check all that apply:	Faculty lack knowledge of how to use audio-based social media platforms for educational purposes
	Residents lack knowledge of how to use audio-based social media platforms for educational purposes
	Program lacks sufficient faculty leadership with expertise in implementing audio-based social media platforms for educational purposes
	Residents do not have the ability to critically appraise audio-based social media platforms for quality and accuracy
	None
Are there any other barriers regarding the implementation of AUDIO-BASED social media that you have encountered?	
VIDEO-BASED SOCIAL MEDIA PLATFORMS	
Which of the following INSTITUTIONAL barriers has your program experienced with regard to implementation of VIDEO-BASED social media into your educational program? Check all that apply:	Institution does not allow for programs to administer video-based social media platforms (e.g., legal department restriction)
	Institution imposes undue regulatory burden on the administration of video-based social media platforms
	Institution restricts access to video-based social media sites on campus internet (e.g., firewall limits computer access)
	None
Which of the following DEPARTMENTAL barriers has your program experienced with regard to implementation of VIDEO-BASED social media into your educational program? Check all that apply:	Department chair does not support or believe in the use of video-based social media for educational purposes
	Insufficient faculty interest in utilizing video-based social media for educational purposes
	Insufficient resident interest in utilizing video-based social media for educational purposes
	Department will not provide adequate funds to support residency-based video-based social media platforms
	Department does not consider video-based social media platforms to be beneficial to the educational curriculum
	Insufficient protected time to develop educational curricula using video-based social media platforms
	Fear of professionalism violations by participating residents and/or faculty
	None
Which of the following TECHNOLOGICAL barriers has your program experienced with regard to implementation of VIDEO-BASED social media into your educational program? Check all that apply:	Insufficient IT support in hosting video-based social media platforms
	Insufficient wireless internet access on campus
	Insufficient support, space, and/or funding for necessary equipment (e.g., computer, microphone, video camera, audio/video editing software)
	None
Which of the following KNOWLEDGE and/or EXPERTISE barriers has your program experienced with regard to implementation of VIDEO-BASED social media into your educational program? Check all that apply:	Faculty lack knowledge of how to use video-based social media platforms for educational purposes
	Residents lack knowledge of how to use video-based social media platforms for educational purposes
	Program lacks sufficient faculty leadership with expertise in implementing video-based social media platforms for educational purposes
	Residents do not have the ability to critically appraise video-based social media platforms for quality and accuracy
	None
Are there any other barriers regarding the implementation of VIDEO-BASED social media that you have encountered?	
OTHER TYPES OF SOCIAL MEDIA PLATFORMS	
Please indicate what other type of social media platform you have used:	
Which of the following INSTITUTIONAL barriers has your program experienced with regard to implementation of OTHER FORMS of social media into your educational program? Check all that apply:	Institution does not allow for programs to administer social media platforms (e.g., legal department restriction)
	Institution imposes undue regulatory burden on the administration of social media platforms
	Institution restricts access to social media sites on campus internet (e.g., firewall limits computer access)
	None
Which of the following DEPARTMENTAL barriers has your program experienced with regard to implementation of OTHER FORMS of social media into your educational program? Check all that apply:	Department chair does not support or believe in the use of social media for educational purposes
	Insufficient faculty interest in utilizing social media for educational purposes
	Insufficient resident interest in utilizing social media for educational purposes
	Department will not provide adequate funds to support residency-based social media platforms
	Department does not consider social media to be beneficial to the educational curriculum
	Insufficient protected time to develop educational curricula using social media platforms
	Fear of professionalism violations by participating residents and/or faculty
	None
Which of the following TECHNOLOGICAL barriers has your program experienced with regard to implementation of OTHER FORMS of social media into your educational program? Check all that apply:	Insufficient IT support in hosting social media platforms
	Insufficient wireless internet access on campus
	Insufficient support, space, and/or funding for necessary equipment (e.g., computer, microphone, video camera, audio/video editing software)
	None
Which of the following KNOWLEDGE and/or EXPERTISE barriers has your program experienced with regard to implementation of OTHER FORMS of social media into your educational program? Check all that apply:	Faculty lack knowledge of how to use social media platforms for educational purposes
	Residents lack knowledge of how to use social media platforms for educational purposes
	Program lacks sufficient faculty leadership with expertise in implementing social media platforms for educational purposes
	Residents do not have the ability to critically appraise social media platforms for quality and accuracy
	None
Are there any other barriers regarding the implementation of OTHER social media platforms that you have encountered?	
NOT USING SOCIAL MEDIA	
What are the reasons that your program has not attempted to utilize any form of social media? Check all that apply:	Insufficient faculty knowledge
	Insufficient resident knowledge
	Insufficient faculty interest
	Insufficient resident interest
	Lack of institutional support
	Lack of departmental and/or chair support
	Lack of technological support
	Lack of funding
	Lack of protected time
	Fear of professionalism violations
	Other
	Prefer not to answer
If there is another reason your program has not attempted to implement social media, please explain.	
IN CONCLUSION...	
Would you find a CORD endorsed white paper beneficial with recommendations on how to overcome identified barriers to entry for the implementation of sociaI media platforms into the emergency medicine residency curriculum?	Yes
	No
Do you have any additional comments?	
